# Editorial Comment: Profile of sexuality and symptoms of lower urinary tract in non-institutionalized elderly

**DOI:** 10.1590/S1677-5538.IBJU.2019.0162.1

**Published:** 2020-02-20

**Authors:** Valter Javaroni

**Affiliations:** 1 Hospital Federal do Andaraí Departamento de Andrologia Rio de JaneiroRJ Brasil Departamento de Andrologia, Hospital Federal do Andaraí, Rio de Janeiro, RJ, Brasil

## COMMENT

The authors conducted a nice survey among elderly in a populacional-base sample, stressing the importance of some urologic under-reported conditions hidden throughout different causes in a population assisted only by public health system (1). They found a huge percentage of elderly men and women with symptoms that could correlate with some diseases, and consequently, motivate search for adequate health assistance in order to reach better quality of life.

Brazil faces a significant increase in the number of older adults. And as the older population grows, this knowledge is usefull for antecipating need for public health resources, expertise and services related to improve quality of life in elderly. Two important aspects that deserve consideration: the definition of sexuality and some limitations of the study. An important indicator of successful ageing is subjective well-being, that is affected by a multitude of factors and is a direct indicator of health outcomes in older adults (2). Sexuality and urinary dysfunctions are major topics for both genders (3).

Sexuality is predominantly recognized only as the sexual act, but specially for elderly has a broader meaning and should include affection, relationships and the erotic and sexual relationship (4). One relevant aspect to consider, in order to stimulate seek for help, also evolves medical education and doctors ability to address this topic (4). Sexual problems are frequent among older adults, but these problems are infrequently discussed with physicians, particularly in women (5).

The questions presented to the interviewees were not shown, as well as some answers. Pathologies such as premature ejaculation, hypogonadism and overactive bladder received unclear diagnostic criteria. The lack of a control group and some variables prevents further analysis of their results. Body mass index, marital status, education categories, physical activity, religion, comorbidities, medicines, prior surgery, social and familial relationships, quality of life impact, Sexualy Transmited Disease knowledge, already proved some influence (4) over authors objectives.

Finnaly, as the number of individuals who tried to seek medical assistance was not researched, perhaps the proposal to reverse the situation should include basic education in health, training of health professionals and also proactive policies that bring patients closer to solutions. But without a doubt, the articule provides additional evidence to support the claim for better public health services that identify and treat men and women with modifiable conditions, contributing to improve well being during ageing.
